# Molecular Interactions for the Curcumin-Polymer Complex with Enhanced Anti-Inflammatory Effects

**DOI:** 10.3390/pharmaceutics11090442

**Published:** 2019-09-01

**Authors:** Yan He, Hongfei Liu, Wangqing Bian, Yue Liu, Xinyang Liu, Shijing Ma, Xi Zheng, Zhiyun Du, Kun Zhang, Defang Ouyang

**Affiliations:** 1 Allan H. Conney Laboratory for Anticancer Research, School of Biomedical and Pharmaceutical Sciences, Guangdong University of Technology, Guangzhou 510006, China; 2 School of Biotechnology and Health Sciences, Wuyi University, Jiangmen 529020, China; 3 Susan Lehman Cullman Laboratory for Cancer Research, Department of Chemical Biology, Ernest Mario School of Pharmacy, Rutgers, The State University of New Jersey, Piscataway, NJ 08854, USA; 4 State Key Laboratory of Quality Research in Chinese Medicine, Institute of Chinese Medical Sciences (ICMS), University of Macau, Macau 999078, China

**Keywords:** Curcumin, polyvinylpyrrolidone (PVP), solid dispersions, molecular modeling, drug-polymer interactions

## Abstract

The molecular interactions between compound and polymeric carriers are expected to highly contribute to high drug load and good physical stability of solid dispersions. In this study, a series of amorphous solid dispersions (ASD) of Curcumin (Cur) were prepared with different polymers by the solvent evaporation method. With the carrier polyvinylpyrrolidone (PVP), the amorphous solid dispersion system exhibits a better solubility and stability than that with poloxamers and HP-β-CD due to the strong drug-polymer interaction. The drug/polymer interaction and their binding sites were investigated by combined experimental (XRD, DSC, FTIR, SEM, Raman, and 1H-NMR) and molecular dynamics simulation techniques. The Curcumin ASD demonstrated enhanced bioavailability by 11-fold and improved anti-inflammatory activities by the decrease in cytokine production (MMP-9, IL-1β, IL-6, VEGF, MIP-2, and TNF-α) compared to the raw Curcumin. The integration of experimental and modeling techniques is a powerful tool for the rational design of formulation development.

## 1. Introduction

**Figure 1:**
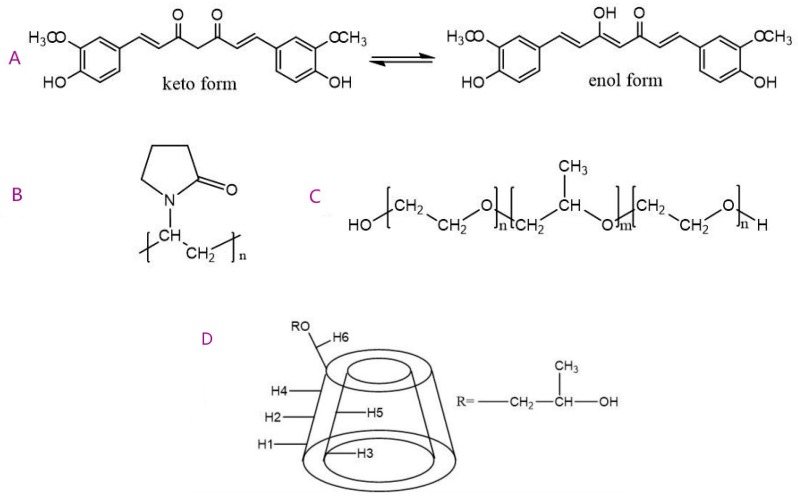
Chemical structures of (**A**) Curcumin (Cur) in keto-enol tautomeric equilibrium; (**B**) PVP K30; (**C**) poloxamer 188; and (**D**) HP-β-CD.

Natural bioactive substances with good anti-inflammatory effects can greatly reduce the risk of complications with low or no toxicity [1,2,3]. Curcumin (1,7-bis(4-hydroxy-3-methoxyphenyl)-1,6-heptadiene-3,5-dione) is a hydrophobic polyphenol derived from the rhizome of turmeric (Curcuma longa), as shown in Figure 1A. Curcumin possesses anti-inflammatory [4], anti-arthritic [5], antioxidant [6], anticancer [7], anti-ischemic [8], and antiproliferative activities [9]. Preclinical and clinical studies also indicate its potential therapeutic value for most chronic inflammation such as lung inflammation, bowel syndrome, and arthritis. Curcumin exhibits potent anti-inflammatory effects targeting the NF-κB, COX-2, 5-LOX, TNF-α, IL-1β, IL-6, IL-8, and MMP-9 factors. Curcumin is nontoxic, even at relatively high doses, with 3.6 g/day above in humans [10]. However, the therapeutic efficacy of Curcumin is limited by its low water solubility, poor stability, rapid metabolism, and low oral bioavailability with only a 1% efficacy in rats [11,12,13,14,15].

Many attempts to improve the bioavailability of Curcumin have been challenged with adjuvants [16]; Curcumin analogues [17]; and improved delivery technologies, such as nanodisks [18], polymeric micelles [19], nanoparticles [20], and polymeric implants [21]. The Curcumin solubility and stability can also be improved by chemical alteration [22]; complexations; interactions; encapsulation with macromolecules; and different carriers such as cyclodextrins (CDs), polymers, and amphiphilic copolymers [23,24]. Curcumin binds to a variety of biopolymers and is known to retain its medicinal activity in the bound states [25]. The formulation of hydrophobic drugs as solid dispersions is a significant area of research aimed at improving their dissolution and chemical stability and, thus, at enhancing the bioavailability. In the solid dispersion system, the solubility of drug can be improved by changing drug crystallinity to an amorphous state and by reducing particle size for better wettability. Different polymers are widely used as vehicles for solid dispersions [26,27] such as polyvinylpyrrolidone (PVP), poloxamers, and HP-β-CD, shown in Figure 1.

Previous research on solubilization of Curcumin involved cationic, anionic, and nonionic polymers including PVP, cyclodextrin, poloxamer, and other adjuvants to improve its dissolution, solubility, stability, and bioavailability. However, it is still a question how it works to improve its anti-inflammatory effects by molecular interaction. Recently, effects were made on the molecular associations, such as hydrogen bonding between the hydroxyl moiety of the drug and carboxylic groups in the carrier and the role of hydrophobic interactions. Deshvir [28] proposed a hypothesis of a novel mechanism of action for the solid dispersion (SD) based on a phenomenon of the hydrogen bond replacement that drifts the drug into the medium from the surface of the SD particles and a rapid release of drug that can be obtained by this system. Maniruzzaman et al. [29] predicted the existence of two possible H-bonding types in the molecular modelling and revealed intermolecular ionic interactions between the active pharmaceutical ingredient (API) amino functional groups and the polymer carboxylic groups through the formation of hydrogen bonding using two model cationic drugs (propranolol HCl and diphenhydramine HCl) with the anionic polymers Eudragit L 100-55 and Eudragit L100. PVP (PVP K30) is an amphiphilic and nonionic homopolymer of N-vinyl-pyrrolidone which contains a carbonic backbone with pyrrolidine rings attached. A segment has the following chemical composition: C6H9NO, which corresponds to the overall composition of the polymer. PVP K30 has low toxicity, strong hydrophilic properties, and physiological tolerance. Polyvinylpyrrolidone showed an important hydrogen bonding with some drugs, such as Rawlinson [30] and Manna [31], to improve dissolution rates of the drug. Poloxamers, commonly known as Pluronics^®^ or Lutrols^®^, are nonionic polymers as the triblock copolymer of the polyoxyethylene (PEO)-polyoxypropylene (PPO)-polyoxyethylene (PEO) structure, which have polymer properties. Ali [32] probed the stoichiometry of the composition which affects the molecular interactions between ibuprofen or ketoprofen and poloxamers within these systems and the molecular interactions that facilitate dissolution rate improvements using solid dispersions.

Current research aims to select different polymers to prepare Curcumin solid dispersions by combined experimental and modeling techniques. The improvement on the stability, bioavailability, and anti-inflammatory activity of Curcumin were studied, and the molecular mechanism and binding sites were further investigated by molecular dynamics simulations.

## 2. Materials and Methods

### 2.1. Materials

Curcumin (Cur) was purchased from Sinopharm chemical reagent Co., Ltd. (Shanghai, China). The polymers were received as gift sample as follows: Polyvinylpyrrolidone (PVP K30, Ashland Co., Covington, KY, USA), poloxamer 188 (Kolliphor^®^ P188, BASF Co., Ludwigshafen, Germany), and hydroxylpropyl-β-cyclodextrin (HP-β-CD, Roquette Co., Ltd., Lesterm, France). All other reagents were of analytical grade.

### 2.2. Methods

#### 2.2.1. Preparation of Solid Dispersions

Solid dispersions were prepared with Curcumin and PVP K30 (Pv), poloxamer 188 (Po), or HP-β-CD (Hp) at weight ratios of 1:1, 1:3, 1:6, and 1:9 respectively for future research by a solvent method. Accurately weighed Cur and polymers were completely dissolved in the minimum amount of absolute ethanol. The solutions were stirred on thermostat magnetic stirrer IT-09A (Yiheng Instruments Co., Ltd., Shanghai, China) for 3 h at room temperature. The solutions were then removed of solvents in the vacuum rotary evaporator RE-2000A (Yarong biochemical Instruments Co., Ltd., Shanghai, China) at 40 °C and dried under vacuum at 40 °C for 3 h. The pulverized powders with 0.18 mm particle sizes were finally obtained.

#### 2.2.2. Characterization

The samples are placed in a closed aluminum pan, and a differential scanning calorimetry pattern was evaluated on the Netzsch DSC 200 F3 Maia^®^ (Gerätebau GmbH, Selb, Germany). The scans were conducted under nitrogen at a flow rate of 20 mL/min, and a heating rate of 20 °C/min was employed from 50 to 270 °C.

Infrared analysis was performed on the Nicolet Nextus 380 FTIR spectrometer (Thermo Electron Corporation, Waltham, MA, USA) with a deuterated triglycine sulfate (DTGS) detector and a KBr beam splitter. Samples were prepared with the KBr disc method (1 mg sample in 100 mg KBr) and scanned in the range of 4000–500 cm^−1^. For each spectrum, 16 scans were performed with a resolution of 4 cm^−1^.

The X-ray diffraction phase formation was characterized with a Ultima IV diffraction meter (Rigaku, Osaka, Japan) with Cu kα radiation (λ = 1.5406 Å) in the range of 3–400 (2θ) at 40 kV and 30 mA. The step width of 0.02 °C was set at the scanning rate of 40/min.

The Raman spectra of Curcumin, polymer, and Curcumin solid dispersion were obtained using an UV laser Raman spectroscopy (LabRAM HR 800, HORIBA Jobin Yvon, Longjumeau France) equipped with a liquid nitrogen cooled Germanium detector. An Nd: YAG laser was used for excitation at 1026 nm with 5 up to 200 mV. All the spectra were an average of 500 scans with 1 cm^−1^ resolutions from 100 to 3000 cm^−1^.

The surface morphology of Curcumin and Curcumin solid dispersion were examined by scanning electron microscopy (SEM, Quanta 400, FEI, Eindhoven, Netherlands). Samples were fixed on a double-sided carbon adhesive tape and sputter-coated with gold under argon atmosphere to render them electrically conductive.

1H-NMR spectra of pure compounds and all prepared solid dispersions were recorded in a Bruker AVANCE III (Bruker, Karlsruhe, Germany) 400 MHz superconducting Fourier. DMSO-d6 was used as the solvent at room temperature.

#### 2.2.3. Determination of Curcumin Concentration

Curcumin content was analyzed by the HPLC system (Shimadzu LC-20AT, Kyoto, Japan) with a diode array detector SPD-M20A with the detection wavelength of 430 nm. The Luna reversed-phase Phenomenex C18 column (Beijing, China) was chosen for detection with ID 4.6 × 150 mm for in vitro solutions and 4.6 × 250 mm for blood samples. The mobile phase was composed of methanol-water (70:30, *v/v*) in vitro and acetonitrile-5% acetic acid (52:48, *v/v*) in vivo at a flowrate of 1 mL/min. The sample injection was 10 μL. All experiments were carried out with dark protection to prevent light-induced degradation of the drug.

#### 2.2.4. Solubility, Dissolution, and Stability Studies

Curcumin and SD powders were added in excess to 10 mL of distilled water, pH 1.2 HCl, 6.8 PBS, and 7.4 PBS respectively in a tube for solubility test. The samples were under constant shaking at 400 rpm for 48 h at 25 ± 2 °C until equilibrium was attained. The sample was filtered through a 0.22-μM MICRO PES filter (Membrana, Obernburg, Germany), and the filtrates were determined by HPLC as mentioned above.

SDs and API equivalents of 10 mg Curcumin were subjected to dissolution studies in 900 mL of pH 1.2 HCl at 37 °C with a paddle stir rate of 50 rpm. Filtered solutions were collected at intervals of 5, 10, 20, 30, 45, 60, 90, and 120 min.

Curcumin could be affected by some metal ions and pH value, thus suppressing its pharmacological activity. The stability of ASD and Cur was evaluated in different conditions of (a) series phosphate buffers (pH 3.0, 6.5, 7.4, 9.0, and 11.0) and (b) 1.0-mM metal ions solutions (FeCl_3_, FeCl_2_, CuCl_2_, ZnCl_2_, and KCl). APIs or SDs with amount to 10 μg of Curcumin were first dissolved in a litter ethanol in the test tube and then supplemented with series phosphate buffers or metal ions solution to get a final of 10 mL. Samples were taken and centrifuged at 10,000 r/min for 5 min at 0, 1, 2, 4, 8, 12, and 24 h. The supernatant was collected after for HPLC determination described above.

#### 2.2.5. In Vivo Bioavailability Studies in SD Rats

The animal studies were carried out according to the guidelines of the local ethical committee on animal experimentation. Male Sprague-Dawley rats (7 weeks old and weighing 240–280 g) were purchased from Laboratory Animal Research Center (Zhengjiang, China) and fasted for 12 h prior to the experiments. The animals were given free access to tap water throughout the experiment period and a normal standard diet 4 h after dosing. The rats were divided into four groups and administered 100 mg/kg oral doses of Curcumin as a suspension in water with 5% (*v/v*) PEG 400. Group 1 (Curcumin suspension in water with 5% (*v/v*) PEG 400, *n* = 3), Group 2 (PV4, 1:4 *w/w*, *n* = 6), Group 3 (PO4, 1:4 *w/w*, *n* = 3), and Group 4 (HP4, 1:4 *w/w*, *n* = 6). About 1 mL of orbit blood was collected at 0 (pre-dose), 0.5, 1, 2, 4, 6, and 8 h post-dose and centrifuged at 16,000× *g* for 10 min. The plasma samples were stored at −20 °C for analysis. After thawing in a water bath at 37 °C, 200 μL of the plasma sample was vortex-mixed thoroughly with 200 μL of acetonitrile for 30 s and then centrifuged at 15,000× *g* for 15 min. Twenty μL of the supernatant was injected into the HPLC system for analysis. A noncompartmental analysis was performed on the BAPP 2.3 software (Center of Drug Metabolism & Pharmacokinetics, Jiangsu, China). The relative bioavailability was calculated between the AUC_0–t_ of Curcumin ASD and API.

#### 2.2.6. Mouse Ear Edema Model and Elisa Assay

Female CD-1 mice (6 weeks old) were used for all experiments. Mice were group housed and provided standard diet. All procedures used in the animal experiments followed the institutional guidelines. The animal experiments were carried out under the protocol (87-115) approved by the Institutional Animal Care and Use Committee (IACUC) of Rutgers University (Permission No. 87-115, 7 January 2014). The mice were randomly divided into 4 groups and each with 3–5 animals. The Curcumin and Cur-PV4 SDs are suspended in water with 10% PEG 400 for doses of 100 mg·kg^−1^. For the ear edema, both ears of CD-1 mice were treated topically with 20 μL of acetone for vehicle control and 1.5 nM phorbol ester (12-O-Tetradecanoylphorbol 13-acetate) (TPA) in acetone. One h before and 1 h and 2 h after TPA treatment, mice were orally given 0.2 mL of (1) vehicle control: water with 10% PEG 400, (2) TPA control: water with 10% PEG 400, (3) Cur: 100 mg·kg^−1^, or (4) Cur-PV4 SDs: 100 mg·kg^−1^. The mice were then euthanized 6 h after the first treatment. Both ear punches (9 mm in diameter) were then taken and weighed. One ear for each group was put into formalin for HE staining, and the other was stored at −80 °C for Elisa assay.

The ears were homogenized in a phosphate buffer and centrifuged at 10,000 rpm for 30 min. The supernatant was used for the IL-1β, MMP-9, IL-6, MIP-2, and VEGF ELISA assays (Biosource, Camarillo, CA, USA). The captureaa antibody was diluted with PBS and used to coat a 96-well plate overnight at room temperature. The plate was then washed, blocked with reagent buffer, and washed again. Thereafter, standards were added to the plate leaving at least one well for background or blank evaluation. The diluted samples (1:3–1:8) were then added to the plate and incubated for 2 h, washed, and incubated with detection antibody for 2 h. Streptavidin conjugated to horseradish peroxidase was then added for 20 min, the samples were washed, and the substrate (H_2_O_2_ and tetramethylbenzidine) was added. After another 20 min of incubation, the stop solution (2 N H_2_SO_4_) was added and absorption was measured with a microplate reader at 450 nm.

#### 2.2.7. Molecular Dynamics Simulation Details

##### Model Building of Drug and Polymer Segments

**Figure 2:**
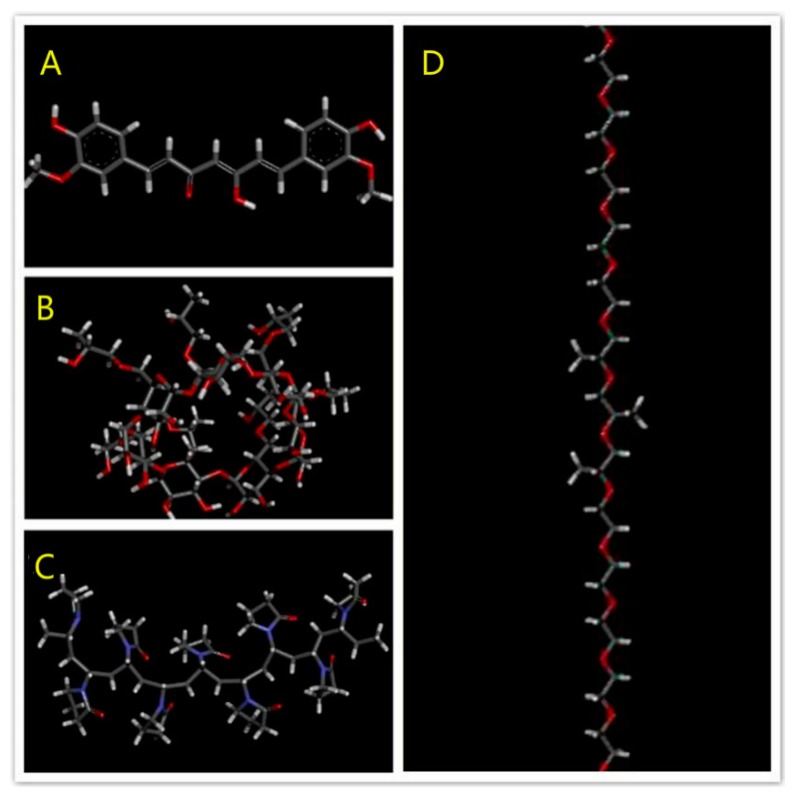
The molecular model of (**A**) Cur, (**B**) polyvinylpyrrolidone (PVP), (**C**) HP-β-CD (HCD), and (**D**) polyxamer 188 (POL).

The molecular model of polyvinylpyrrolidone K30 (PVP), polyxamer 188 (POL), and HP-β-CD (HCD) polymer segments and Curcumin were built by Discovery Studio Visualizer 3.1. The structures of all molecules were optimized with a fast, Dreiding-like force field by Discovery Studio Visualizer. The model of PVP contained 9 repeating units of vinylpyrrolidone. The model of POL was composed of a central chain with 3 repeating units of propylene oxide flanked by 2 chains with 8 repeating units of ethylene oxide. Figure 2A–D showed the molecular models of the Cur molecule and the PVP, HCD and POL polymer segments, respectively.

##### The Molecular Dynamics (MD) Simulation

Simulation details for solid dispersions of PVP and POL were as follows. The molecular dynamics (MD) simulations used the AMBER14 software package with the general AMBER force field (gaff) [1] for the drug and polymer: PVP and POL. According to the experimental condition, the molar ratio of number Cur:PVP was 1:3 and the ratio of Cur:POL was 1:4. The Packmol program was used to build the initial structure of the systems by packing molecules in defined regions of space. For PVP/Cur system, it contained 5 drug molecules and 15 PVP chains, while the POL/Cur system had 5 drug molecules and 20 POL polymer chains. Both systems were input into the LEAP module in AmberTools. For the simulation of solid dispersion, the simulated annealing method [2] was used to mimic the hot melt preparation method of solid dispersion in the experiments [3,4,5]. In the minimization procedure, the structures of drug-polymer were subjected to 1000 steps of steepest descent minimization followed by 1000 steps of conjugate gradient minimization. After minimization, the system was gradually heated from 0 to 500 K in 200 ps and then kept at a temperature of 500 K for 800 ps to equilibrate the systems. After that, the system was quickly cooled down from 500 to 300 K in 200 ps, and finally, the systems were kept at a temperature of 300 K for 800 ps s for equilibration. In the simulation, the Langevin dynamics was used to control the temperature using a collision frequency of 2 fs and the nonbonded cutoff distance was 10.0 Å.

For the HCD/Cur system, the stable binding model from docking was used as the starting structure of MD simulation. In our modeling, the AutoDock Tools package (version 1.5.6) and AutoDock Vina (version 1.1.2) [6] were used to perform docking studies [7]. In docking simulation, Cur was used as a ligand while HCD was used as a receptor. The molar ratio of Cur:HCD was 1:1. After docking, a water cube of 10 Å thickness was added to solvate the system with the TIP3P water model in the LEAP module of AmberTools. After the minimization, 50 ns MD simulations were performed. The detailed simulation protocol was similar to our previous simulation researches [3,4,5].

## 3. Results and Discussion

### 3.1. Physicochemical Characterization

#### 3.1.1. Solubility, Dissolution, and Stability

**Figure 3:**
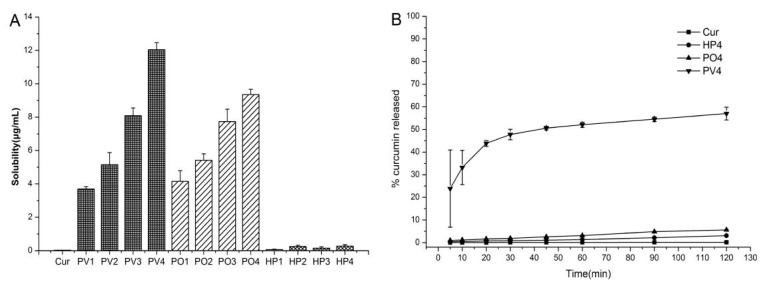
(**A**) Solubility in distilled water and (**B**) dissolution profiles in distilled water (pH 6.8) of solid dispersions. Data are expressed as mean ± S.D. (*n* = 3).

The polymer solid dispersions were tested with four different ratios of Cur and PVP (PO or HP) as 1:1, 1:3, 1:6, and 1:9 in this paper. Cationic and nonionic polymers improved the solubility of Curcumin owing to its affinity and miscibility. In Figure 3A, Curcumin formulation with PV4 (1:9 ratio) showed the best solubilization performance among all formulations. The curves in Figure 3B show PV4 groups exhibited the best dissolution behavior with considerably increased speed and extension. The percentage of PV4 Curcumin released was approximately 33% for the SD in the initial 10 min, whereas the release of unformulated Cur was slow with less than 1% within 120 min. Thus, the cationic polymer PVP was the best choice for improving the solubility and dissolution behaviors of Curcumin, by the conversion from crystalline state to amorphous state.

**Figure 4:**
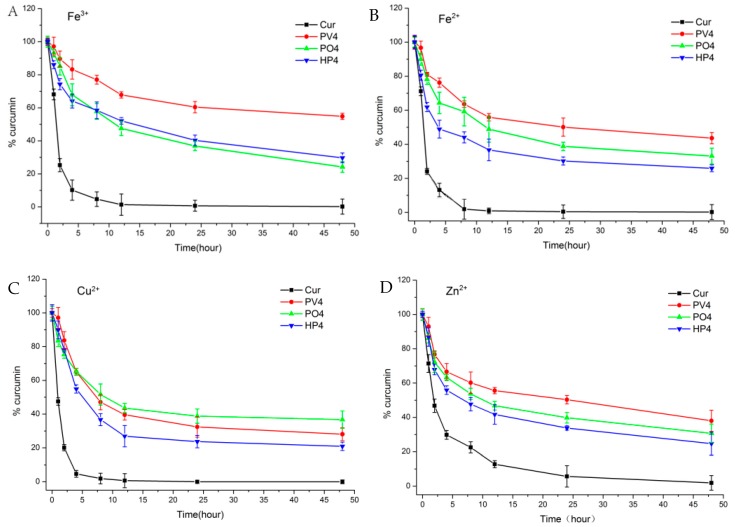

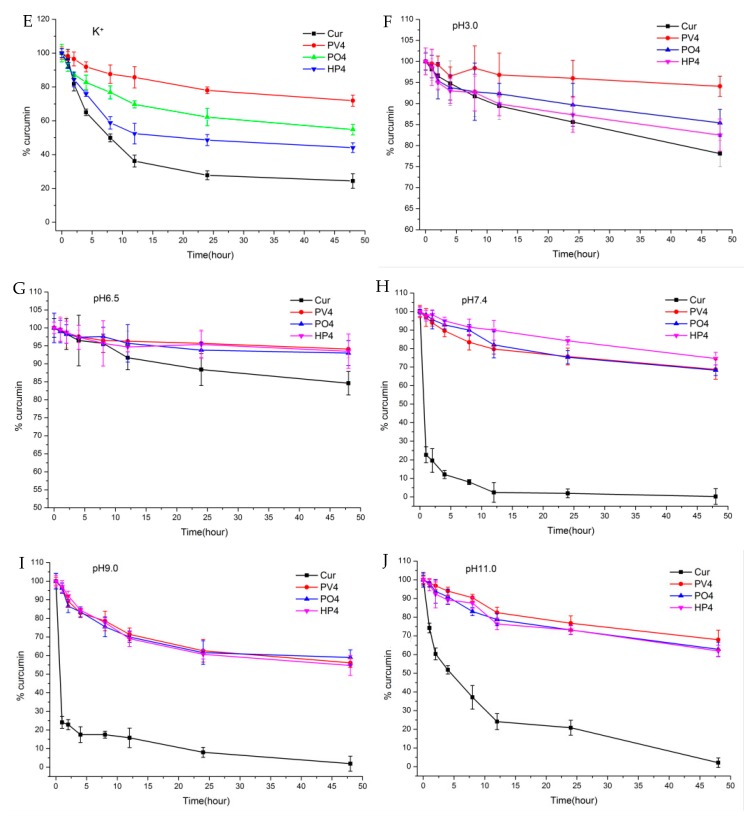
(**A**–**E**) The stability of Curcumin and Curcumin solid dispersion in metal ions solution and (**F**–**J**) phosphate buffer solutions of different pH values.

Solid dispersions have greatly improved the tolerance of these metal ions by protecting Cur from chelating with some metal ions to enhance their stabilities. The metal ions had great influence on the stabilities of all groups as shown in Figure 4A. The Curcumin was degraded significantly with almost no Curcumin found after 24 h for API solutions with Fe^3+^, Fe^2+^, Cu^2+^, and Zn^2+^. Solid dispersions, especially PVP ASD, have greatly improved the tolerance of these metal ions and slowed down the degradations.

As shown in the Figure 4B, Curcumin is stable in weak acidic condition (pH 3.0 and 6.5) and is unstable under neutral or basic pH conditions with a fast degradation at pH 7.4, 9.0, 10.0, and 12.0 and less than 1% Curcumin detected after 48 h. As for the tested groups, PV4 had the highest stability and tolerance to Curcumin in different acidity and alkalinity conditions, followed by HP4.

Overall, all three excipients can improve the tolerance and stability of Curcumin to metal ions and pH, with PV4 group better than that of other two carrier groups. PVP and Curcumin form hydrogen bonds at the hydroxyl position of phenol, and the carbonyl group of PVP forms positive and negative ionic bonds with the Curcumin bicarbonyl group, showing a more stable state. Both PO and HP formed hydrogen bonds at the hydroxyl site of phenol, reducing the protection of the dicarbonyl structure. The hydrogen bond within the PO and HP ASD groups is relatively weak because the single chain of PO is relatively long. The HP4 was less stable because HP-β-CD locked one side of phenolic hydroxyl group on Curcumin into the cavity with the dicarbonyl group exposed outside. Poloxamer belongs to nonionic polymer with a long chain. There are two hydroxyl groups at both ends and two phenolic hydroxyl groups on Curcumin to form hydrogen bonds. However, this chain is long and easy to break when dispersed in solution, so its stability is relatively low.

#### 3.1.2. Other Characterizations

XRD, DSC, FTIR, SEM, Raman, and 1H-NMR were used to characterize the interaction forms and binding sites between Curcumin and PVP, poloxamer, and HP-β-CD.

**Figure 5:**
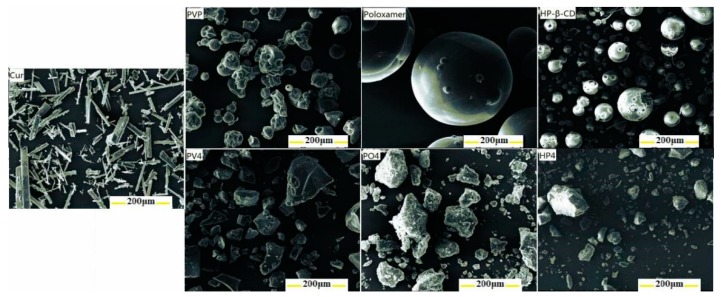
SEM images of the pure Curcumin, surfactant, and Curcumin solid dispersion.

No observable Curcumin with columnar crystalline morphology feature was found in the solid dispersions, and the Curcumin was homogeneously dispersed into the carriers in the amorphous form at the molecular level as shown in the Figure 5.

**Figure 6:**
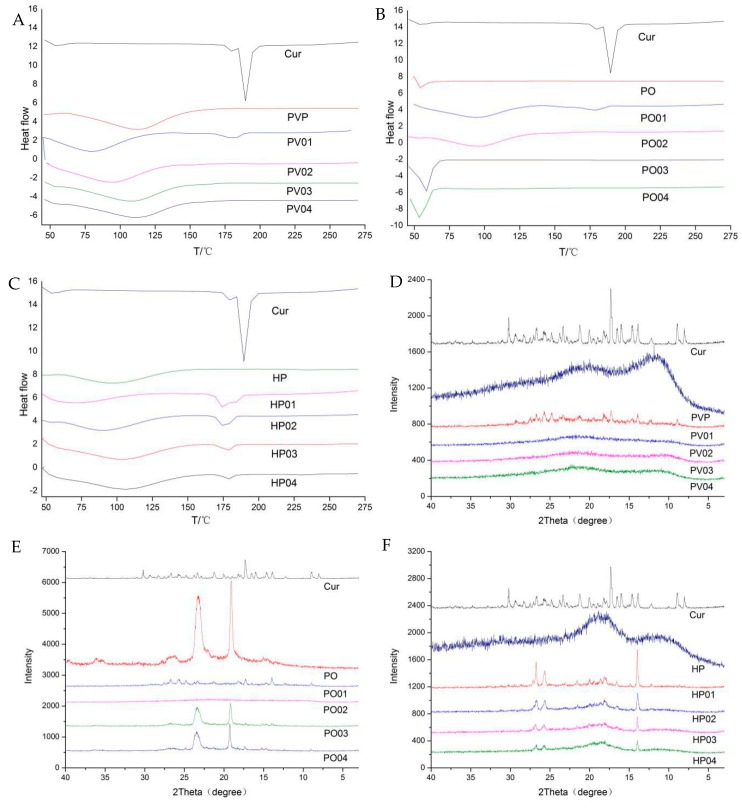

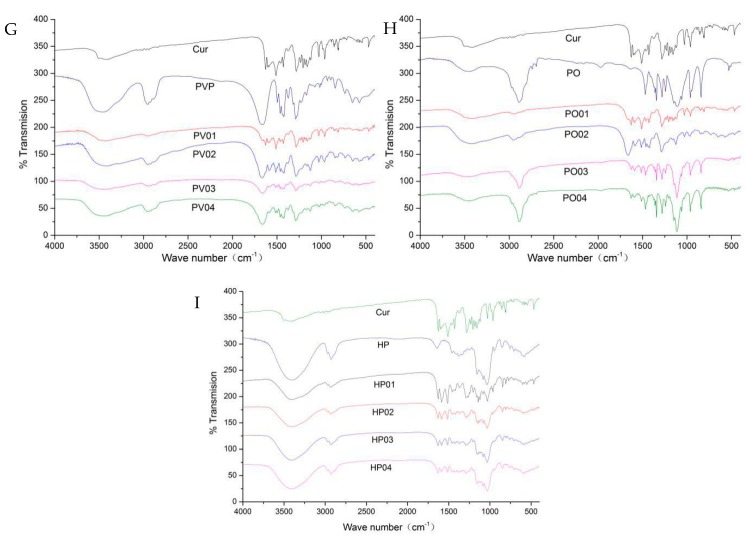
(**A**–**C**) DSC, (**D**–**F**) X-ray, and (**G**–**I**) FTIR spectrum of Curcumin, polymers (various carriers), and Curcumin solid dispersions.

The DSC thermograms (Figure 6A–C) show that the endothermic peak of Curcumin is at 189.59 °C. There are absorption peaks of water evaporation for amorphous PVP and HP-β-CD between 50 °C and 125 °C, while there is only a melting peak for poloxamer with a low melting point. At the weight ratio 1:1 of Curcumin to PVP, the melting characteristic peaks of Curcumin still existed, indicating the microcrystalline form of Curcumin in the PV1. When the ratio increased to 1:3, 1:6, and 1:9, the melting peaks disappeared, hinting that Curcumin was dispersed uniformly in PVP in the amorphous or molecular states. The low melting point of poloxamer is about 54 °C during the heating process. PO01 and PO02 exhibited no melting endotherm of crystalline poloxamer and significant water evaporation, as well as the disappearance of the melting peak of Cur. However, PO03 and PO04 exhibited larger the melting endotherm over 50 °C. The possible reason is that poloxamer and Cur formed amorphous solid dispersion at the ratio 1:1 (PO01) and 1:3 (PO02), while poloxamer and Cur formed an eutectic mixture at the ratios 1:6 (PO03) and 1:9 (PO04). Further research is necessary for detailed mechanism. In addition, Curcumin and HP-β-CD may form the complex.

In the XRD spectroscopy (Figure 6D–F), the distinguishable crystal diffraction peaks of Curcumin have completely disappeared in solid dispersion. The Curcumin is dispersed in the amorphous form in PVP, achieving a highly dispersed state. With the increase of PO and HP-β-CD -Cur, Curcumin diffraction peaks gradually disappear but still existed even at the upper ratio in this study.

The changing peak intensity or shift in the wave number was studied by FTIR spectroscopy (Figure 6G–I). As for Curcumin, the peak 3417 cm^−1^ indicated the presence of OH, the peak of 1627 cm^−1^ was mixed ν (C=C) and ν (C=O) characters and another band at 1602 cm^−1^ caused by the symmetric aromatic ring stretching vibrations ν (C=Cring). The 1510 cm^−1^ peak was assigned to the ν (C=O), while enol the C–O peak was at 1283 cm^−1^ and the C–O–C peak was at 1029 cm^−1^. Curcumin often forms hydrogen bonds with solubilizing carriers at hydroxyl sites in phenolic, methoxy, or enol structures. The important bands of PVP at 2954 cm^−1^, 1658 cm^−1^, and 1289 cm^−1^ denoted respectively the stretching vibration of C–H, C=O, and C–N. The broad band at 3451 cm^−1^ was due to the presence of water confirming that the broad endothermic peak was detected in the DSC. The characteristic absorption peaks of poloxamer around 3450 cm^−1^, 2888 cm^−1^, and 1111 cm^−1^ were attributed to O–H, C–H, and C–O–C stretching vibrations. The characteristic peak of HP-β-CD was at 3406 cm^−1^ for the OH group of stretching vibrations, and the band at 2926 cm^−1^ was assigned to the CH and CH_2_ groups, as well as some other prominent peaks at 1639 cm^−1^ (H–O–H bending), 1155 cm^−1^ (C–O), and 1034 cm^−1^ (C–O–C). The O–H stretching vibration at 3417 cm^−1^ disappeared in PV4, PO4, and HP4 solid dispersion. The C=O peak owing to Curcumin shifted visibly in PV4 and PO4. Curcumin was enwrapped into the cavity of HP-β-CD, and the C=O peak also shifted significantly. Thus, there was an intermolecular hydrogen bond between Curcumin and the three carriers, and the carbonyl group of pyrrolidone in PVP was proton acceptor forming hydrogen bonding with Curcumin potentially.

**Figure 7:**
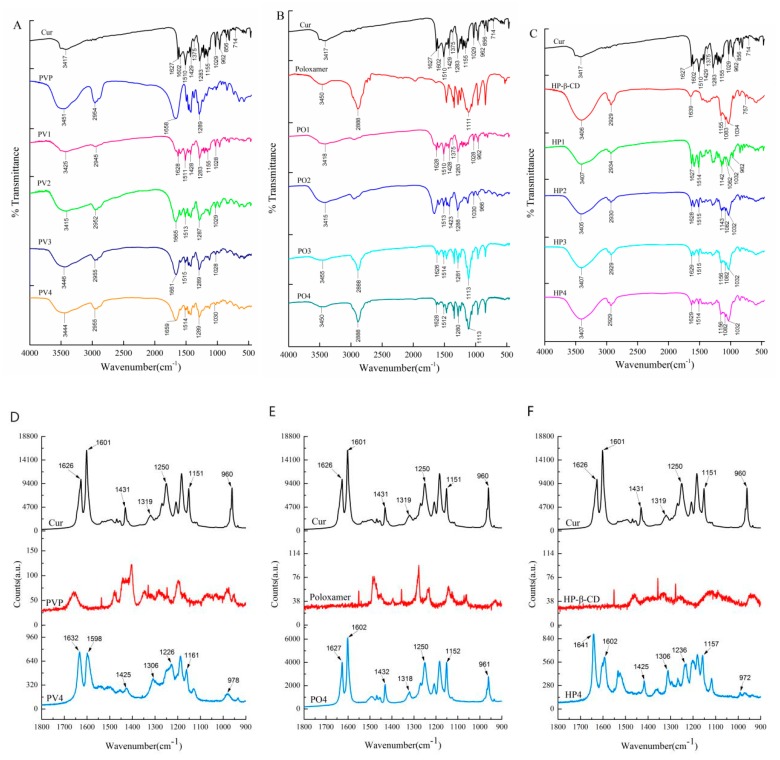
The (**A**–**C**) FTIR and (**D**–**F**) Raman spectrum of Curcumin and Curcumin solid dispersion.

The Raman spectrum peak of Curcumin and its solid preparations are shown in the following Table 1 and Figure 7 which is consistent with the FTIR results. The Curcumin dicarbonyl group has no absorption peak from 1650 cm^−1^ to 1800 cm^−1^. The structure of Curcumin is conjugated with C=O and C=C (CO–C=C), with its characteristic peak shifting to 1626 cm^−1^, which proves that Curcumin exists as an enol structure. The peak 1601 cm^−1^ represents the stretching vibration absorption peak of C=C on the benzene ring, while the 1250 cm^−1^ represents the deformation vibration of CCH on the benzene ring and the deformation vibration of enol C–OH. In the PV4 group, the characteristic peak of C=O conjugated with C=C (CO–C=C) shifted to 1632 cm^−1^ with decreased intensity. The stretching vibration of C=C on the benzene ring shifted from 1601 cm^−1^ to 1598 cm^−1^. The deformation vibration of CCH on the benzene ring and enol C–OH disappeared. It may be that the carbonyl group of PVP and the Curcumin phenolic hydroxyl group and enol C–OH formed intermolecular hydrogen bonds combined with FTIR analysis. In the PO4 group, 1626 cm^−1^ and 1601 cm^−1^ migrated to 1627 cm^−1^ and 1602 cm^−1^ respectively, which confirmed an intermolecular hydrogen bond between the hydroxyl group at the end chain of poloxamer and two phenolic hydroxyl groups, enolic C–OH and the methoxyl groups of Curcumin. In HP4, the Curcumin benzene ring migrates from 1626 cm^−1^ to 1632 cm^−1^ with and decreased intensity and the enol structure of Curcumin migrated to 1641 cm^−1^ at 1626 cm^−1^ with and changed intensity. It shows that the Curcumin benzene ring enters the cavity of HP-β-CD and forms a steric hindrance effect on enol C–OH.

The combination of Raman spectroscopy and infrared spectroscopy can better analyze the molecular binding sites of Curcumin to the carrier. PVP, with the helical polymerized straight chain structure, encapsulated Curcumin by intermolecular hydrogen bonds with the phenol hydroxyl and enol hydroxyl group of Curcumin. The lactam of PVP which did not interact with Curcumin was outward bound and had good hydrophilicity, so the water solubility of Curcumin was greatly improved. Poloxamer forms hydrogen bonds with Curcumin in H or OH at the end of the chain, but there is still a contact surface between Curcumin and water, and its water solubility is lower than that of PV4. HP-β-CD encapsulates the phenyl ring of Curcumin in its cavity, but the fat chain in the middle part of Curcumin may have a larger contact surface with water, so its water solubility is the lowest.

#### 3.1.3. 1H-NMR Analysis

**Figure 8:**
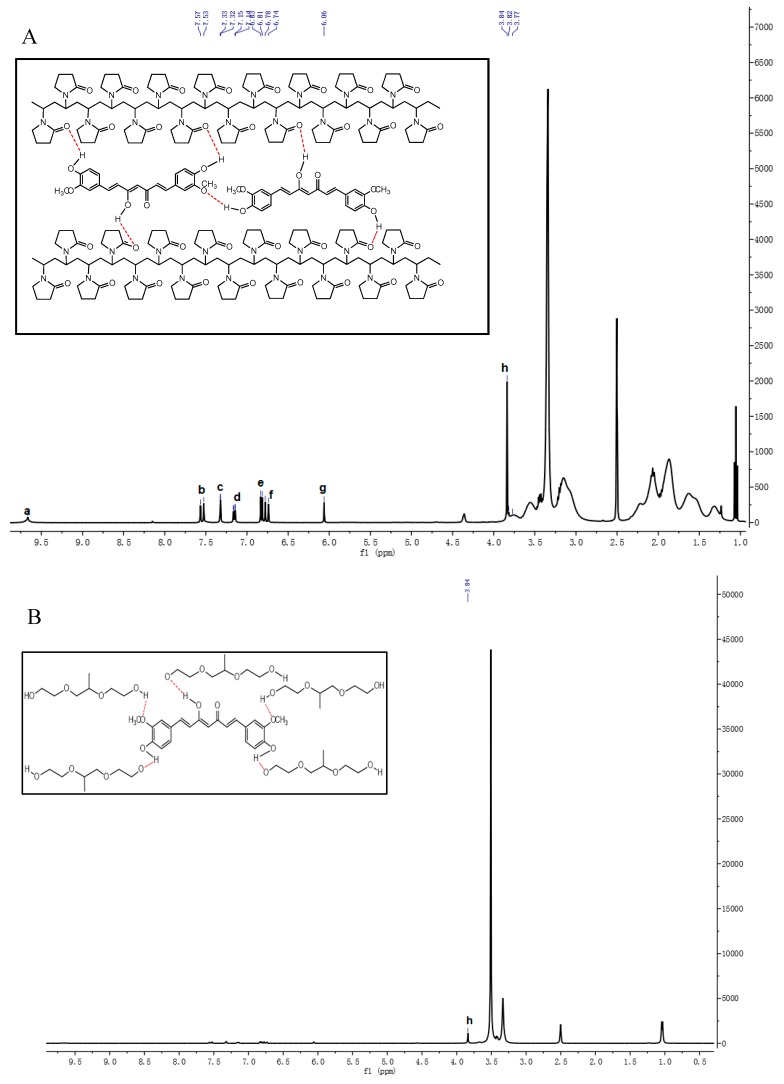

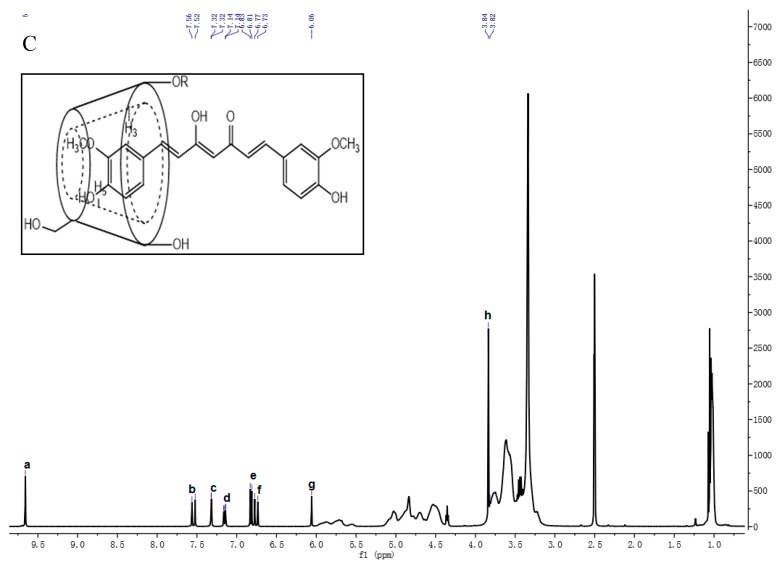
The 1H-NMR spectrum and intermolecular interaction of (**A**) PV4; (**B**) PO4; and (**C**) HP4.

1H-NMR experiments were performed to evaluate the chemical shifts (∆δ) of hydrogen atoms in the molecular structure of Curcumin, polymer, and their solid dispersions to clarify the interaction form and site of Curcumin and polymer in molecular structure as shown in Figure 8A. PVP significantly improve the water solubility of Curcumin better than Poloxamer and HP-β-CD by intermolecular interaction and formation of ionic bonds and hydrogen bond. 

The chemical shift of the phenolic hydroxyl group of Curcumin moves to high field, which further confirms the formation of hydrogen bond in PV4. Molecular dynamic simulation studies have proposed that PVP adopts a double spiral structure with the backbone protons (H-1 and H-2) inside and pyrrolidone carbonyls outside exposed to the water. Curcumin is wrapped on the hydrophobic side of PVP, while hydrogen bonds are formed on the hydrophilic side to increase water solubility in PV4. The chemical shifts of methyl and methylene in the hydrophobic long chain of PVP have changed in PV4, which means that H-1 and H-2 are closer to Curcumin in the formation of solid dispersion. The chemical shift happens to other hydrophilic regions (H-3, H-4, and H-5) of PVP and enhanced the interaction with Curcumin by hydrogen bonding to facilitate Curcumin surrounded by hydrophobic region. The N+ on PVP attracts H-3 protons to reduce the H-3 electron cloud density and forms ionic bonds with Curcumin dicarbonyl oxygen anions between Curcumin and PVP. 

Poloxamer is a nonionic PEOn-PPOm-PEOn copolymer with hydrophilic poly(ethylene oxide) (PEO) blocks and hydrophobic poly(propylene oxide) (PPO) blocks in a triblock structure. The three characteristic chemical shifts all migrated in poloxamer, including PPO methyl (1.029 ppm), PPO methylene (3.327 ppm), and PEO methylene (3.508 ppm), during the formation of PO4. Combined with the previous characterization, the H or OH at the end of the chain of poloxamer forms hydrogen bonds with the phenol hydroxyl and methoxy groups of Curcumin and then encapsulates Curcumin into the center to form polymer micelles for solubilization purposes. The hydrogen bonds are mainly formed at both ends of PEO but are easy to break due to the long intermediate polymer chain. Poloxamer has a similar effect on other insoluble drugs, improving the solubility of insoluble drugs [33]. 

1H-NMR showed the change of chemical shifts of H-3 and H-5 protons in HP-β-CD, which proved that insoluble Cur entered the cavity of HP-β-CD to form the inclusion complex [34]. In HP4 Curcumin, the hydrogen of methoxy group on the benzene ring moves to high field to produce the shielding effect, which makes the chemical shifts of H-3 and H-5 in HP-β-CD move to high field. Furthermore, the high electronegativity of the oxygen atoms of methoxy (OCH_3_) groups contributed to the chemical shifted of H-1, H-2, H-4, and H-6. The specific Curcumin peaks between 6.50 and 7.60 ppm (aromatic rings) were shifted in HP4, which further confirms Curcumin inside HP-β-CD cavity. However, the chemical shifts of Curcumin hydroxyl group with lower peak intensity still exist in the 1H-NMR spectrum of HP4, suggesting that only one side of the benzene ring of Curcumin may enter the cavity.

#### 3.1.4. Modeling Results

**Figure 9:**
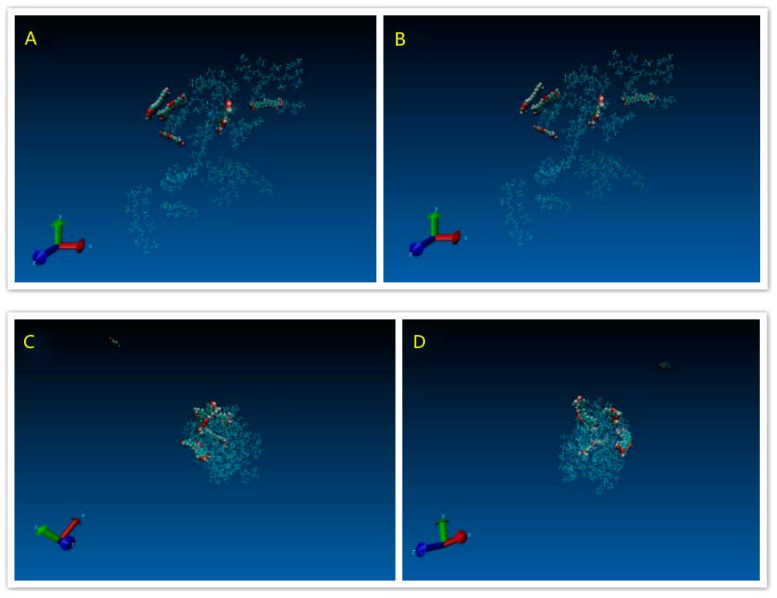
Snapshots of PVP/Cur solid dispersion systems: (**A**) Initial structure system; (**B**) structure after minimization procedure; (**C**) system structure after 4 ns molecular dynamics (MD); and (**D**) system structure after 4 ns MD (different side).

**Figure 10:**
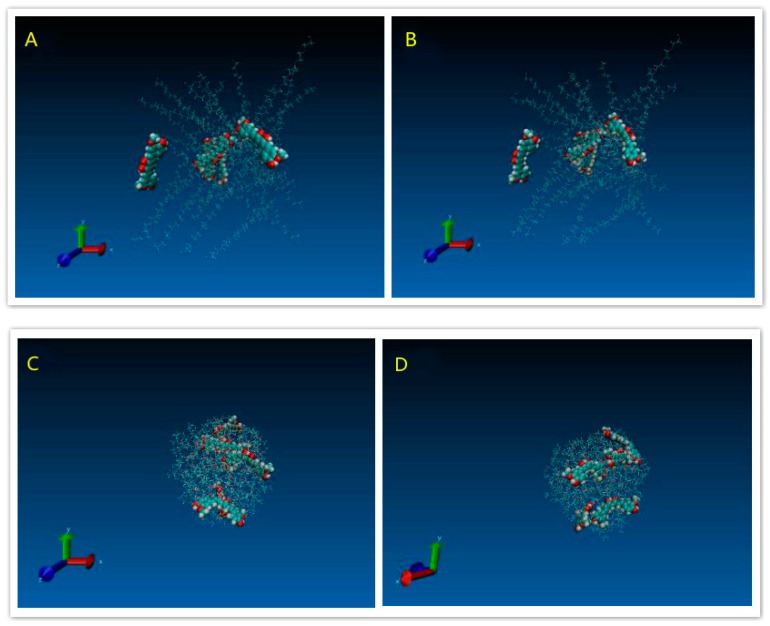
Snapshots of POL/Cur solid dispersion systems: (**A**) Initial structure system; (**B**) structure after minimization procedure; (**C**) system structure after 4 ns MD; and (**D**) system structure after 4 ns MD (different side).

Figure 9A indicated the initial structure of the PVP/Cur solid dispersion system. After energy minimisation and simulation, the state of the whole system was showed like the Figure 9B. Figure 9C,D showed that the polymer, which bended and folded irregularly, formed into the random coils. For the PVP/Cur system, four Curcumin molecules are attached to the PVP coils, while still one drug molecule was free of the system. Figure 10A–D indicated the POL/drug system, which was similar to the PVP/drug system. All five drug molecules in the POL/Cur system were strongly stuck at the surface of POL coils. These results may give us an explanation of the better performance of PVP system in the dissolution experiments because Curcumin molecules were easily released from the system in the water.

**Figure 11:**
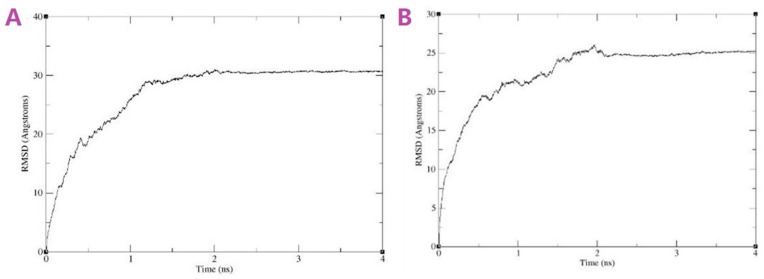
RMSD versus time of (**A**) PVP polymer segment and Cur complex and (**B**) POL polymer segment and Cur complex in 4 ns simulation.

**Figure 12:**
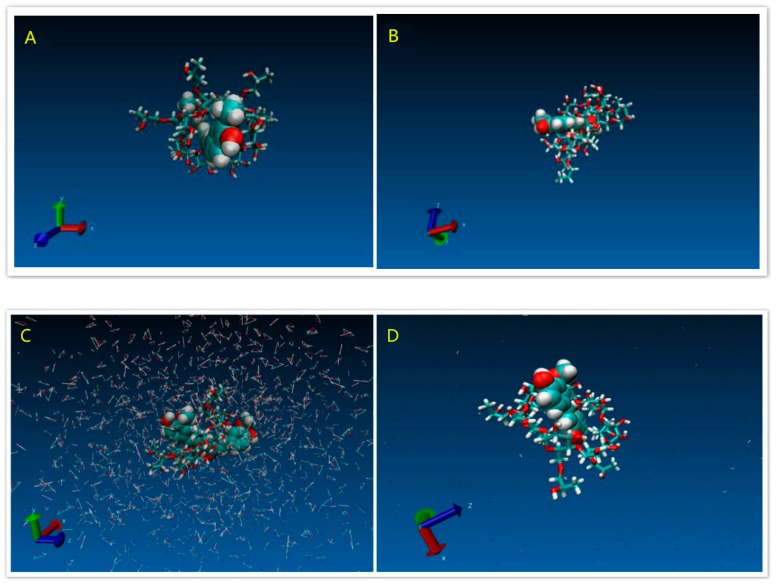
Snapshots of the HCD/Cur inclusion complex: (**A**) HCD/Cur initial structure system; (**B**) HCD/Cur structure after 50 ns MD; (**C**) initial structure in solvent system; and (**D**) system structure after 50 ns MD in the solvent system.

Figure 11A,B revealed the Curve of RMSD versus time of both systems, which showed the equilibrated systems after 2 ns. Figure 12A showed the initial structure of the HCD/Cur system. Figure 12C showed the initial structure in the solvent system. These snapshots indicated the unstable state of the complex. In the simulation, the structure of HCD/Cur complex changed much at the first 6 ns. After that, the HCD/Cur complex became stable. The Figure 12C,D showed the stable structure with or without the solvent at 50 ns. In the simulation, the Cur totally inserted into the cavity of HCD which was in agreement with the experimental part. Therefore, the MD simulation could help us to understand the molecular mechanism of drug/polymer complex.

### 3.2. Oral Bioavailability In Vitro

**Figure 13:**
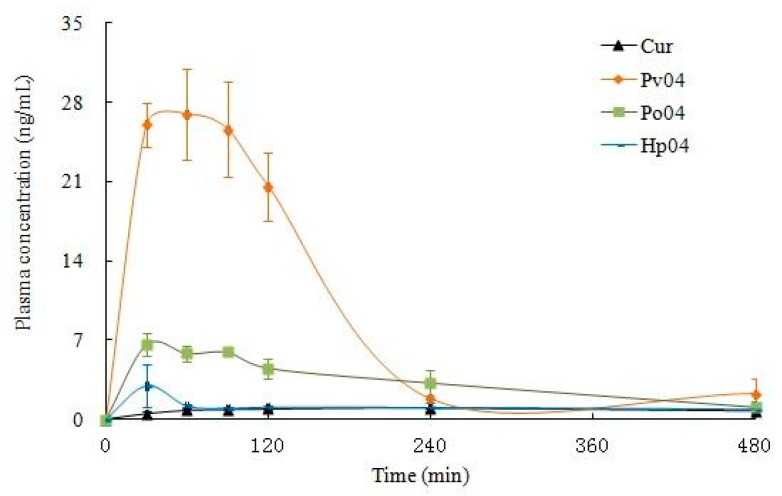
Plasma Curcumin Curve after a single oral dose of PV4 and Curcumin (*n* = 3) in male SD rats: Male Sprague-Dawley rats (7 weeks old and weighing 240–280 g) were used. Rats were fasted for 12 h prior to the beginning of experiments. All groups received Curcumin: Curcumin, PV04, HP04, and PV04 suspension in water with 5% (*v/v*) PEG 400, *n* = 3. One mL of eye blood was collected at 0.5, 1, 2, 4, 6, and 8 h post-dose.

The retention time of Curcumin was 10 min with a good separation to plasma constituents (Figure 13). The standard Curve of Curcumin in plasma was linear over the concentration range of 0.1–50 μg·mL^−1^ examined. The regression equation for Curcumin was y = 32410x − 5179.0 (r = 0.9996), where y was peak area and x was the plasma concentration of Curcumin. The precision was measured at three different concentrations (0.5, 10.0, and 20.0 μg·mL^−1^). The relative standard deviation (RSD) was less than 2.0%. The mean extraction recovery was 77.8 ± 1.9%, 81.4 ± 1.8%, and 79.5 ± 0.9% at 0.5, 10.0, and 20.0 μg·mL^−1^, respectively. The limit of quantization (S/N = 10) was 300 ng, and the limit of detection (S/N = 3) of Curcumin was 100 ng in plasma.

We measured levels of Curcumin in plasma of Sprague-Dawley rats orally dosed with unformulated Curcumin and ASD Curcumin. Figure 13 shows the time course of Curcumin concentrations in plasma after a single oral dose of either unformulated Curcumin or ASD Curcumin, and the pharmacokinetic parameters are summarized in Table 2. Relative bioavailability of calculated PV4, PO4, and HP4 demonstrated 11, 4, and 1.3-fold increases in AUC_0–t_ and 28, 6, and 3-fold increases in C_max_ concentration respectively compared to unformulated Curcumin, which indicated that the solid dispersion had a certain effect on the oral absorption enhancement of Curcumin.

### 3.3. Anti-Inflammatory Efficacy on TPA-Induced Mouse Ear Edema

**Figure 14:**
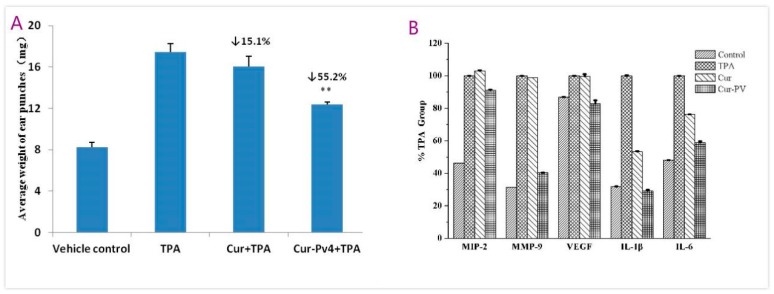

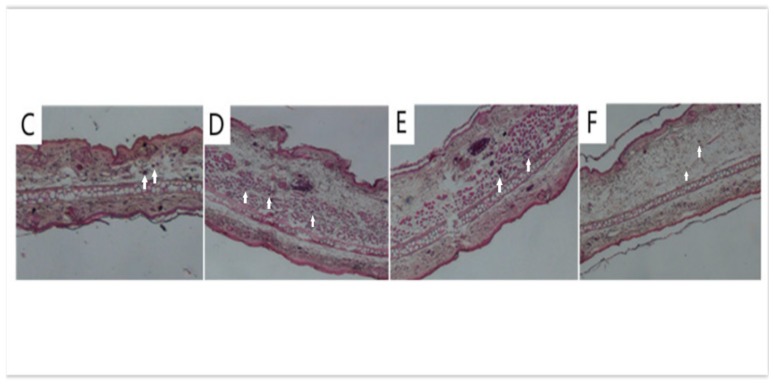
(**A**) Effect of Curcumin and Cur-PV4 on the weight of ear punches, with (**B**) immunohistochemical staining on IL-1β, IL-6, MMP-9, MIP-2, and VEGF and H&E staining for histological changes of TPA-induced mouse ears: (**C**) Acetone, (**D**) TPA, (**E**) Curcumin, and (**F**) Cur-PV4. Magnification 200× (3). Each value represents the mean ± S.D. (*n* = 3).

As shown in the Figure 14A, there is a significant difference in ear weights between the Curcumin and Cur-PV4 (** *p* < 0.01). Histological sections of the ears submitted to a single TPA application (Figure 14D) revealed a significant increase in the infiltrating inflammatory cells when compared to the acetone-treated ears (A, vehicle). As described in Figure 14E,F, treatment of mice with Curcumin-TPA (Figure 14E) and Cur-PV and TPA (Figure 14F) decreased the number of infiltrating inflammatory cells to some extent. Representative infiltrating inflammatory cells are indicated by arrows. The mice ears punches were homogenized and t analyzed by ELISA for the levels of MMP-9, IL-1β, IL-6, VEGF, and MIP-2 (Figure 14B). Differences between Curcumin and Cur-PV were significant (*** *p* < 0.001) for decreasing TPA-induced MMP-9, IL-1β, and IL-6 levels and not significant for decreasing TPA-induced VEGF and MIP-2 levels. Curcumin shows good anti-inflammatory activities against a vast array of molecular targets for the prevention and treatment of various chronic diseases [1]. The water-soluble Curcumin formulation effectively improve the anti-inflammatory effect of Curcumin here.

## 4. Conclusions

The present study confirmed that the blends of PVP, poloxamer 188, and HP-β-CD are appropriate carriers for the preparation of Curcumin SDs by the solvent evaporation method. DSC, XRD, FTIR, Raman, SEM, and 1H-NMR were used to confirm the transformation of Curcumin from the crystalline to the amorphous state. PVP was better than poloxamer 188 and HP-β-CD on improving the solubility, stability, and bioavailability of Curcumin. Further study indicated that Cur-PVP effectively improved the anti-inflammatory effect of Curcumin. Differences between Curcumin and Cur-PV were significant (*** *p* < 0.001) for decreasing TPA-induced MMP-9, IL-1β, and IL-6 levels. Our studies proved that Cur-PVP solid disperison is a promising and convenient method for improving oral bioavailability and anti-inflammatory effects of Curcumin. With the enhanced curcumin solubility, stability, and absorption, many chronic inflammatory diseases will be at the forefront as promising targets for curcumin therapy.

## Figures and Tables

**Table 1:** The Raman spectrum peak of Curcumin and its solid preparations.

Cur (cm^−1^)	PV4 (cm^−1^)	PO4 (cm^−1^)	HP4 (cm^−1^)	Peak Assignment
1626	1632	1627	1641	Expansion vibration of C=C and C=O on enol structure
1601	1598	1602	1602	Telescopic vibration of C=C on the benzene ring
1431	1425	1432	1425	Planar vibration of C–C, C–CH, and C–OH on the benzene ring
1319	1306	1318	1306	C–CH planar vibration of C^10^ and C^11^ on the enenol structure
1250	1229	1250	1236	Deformation vibration of CCH on the benzene ring and C–OH of enenol
1151	1161	1152	1157	Deformation and vibration of C–CH on the benzene ring, C–OH, and C=CH
960	978	961	972	Planar vibration of CCH on the benzene ring

**Table 2:** Pharmacokinetic parameters after oral administration of Curcumin and Curcumin solid dispersion calculated by a non-compartmental method.

Parameters	$\bar{X}\pm \mathrm{SD}\;(\mathrm{Cur})$	$\bar{X}\pm \mathrm{SD}\;(\mathrm{PV}4)$	$\bar{X}\pm \mathrm{SD}\;(\mathrm{PO}4)$	$\bar{X}\pm \mathrm{SD}\;(\mathrm{HP}4)$
T_max_ (h)	1.33 ± 0.00	1.17 ± 0.58	1.00 ± 0.50	0.50 ± 0.00
C_max_ (ng/ mL)	1.07 ± 0.18	28.63 ± 5.50	6.64 ± 1.00	2.98 ± 1.89
AUC_0–t_ (ng/mL·h)	6.72 ± 1.25	74.76 ± 21.61	26.46 ± 1.53	8.73 ± 2.72
T_1/2_ (h)	10.17 ± 0.00	1.17 ± 0.21	2.80 ± 0.21	4.50 ± 3.12
F		11.13	3.94	1.30

Data are expressed as mean ± S.D. (*n* = 5). Each value represents the mean ± S.D. (*n* = 5).
